# Let Us Have Peace

**Published:** 1887-05

**Authors:** 


### "LET US HAVE PEACE."

To an impartial observer it would appear that the question of Dentistry *versus* Medicine has been more than sufficiently discussed. From the commencement, the controversy has assumed an acrimonious and personal character which was quite uncalled for, and the feeling is becoming more bitter with each rejoinder. He is said to be a foolish man who quarrels with his bread and butter, and the man who foments an insurrection in his own domestic circle is certainly no wiser. Medicine, to say the least, is our closest relative and nearest neighbor, and what is to be gained by provoking an altercation with it is not readily apparent. So far, our mother—or sister, according as we may view the relationship—has prudently refrained from noticing the chip which we are so provokingly carrying on our shoulder, and has left the brawling to us. The controversy can have no decisive end, and we cannot see what good can possibly grow out of it. Certainly, it does not tend to elevate our professional status, or advance professional interests.

To our apprehension, the whole discussion grows out of a misunderstanding of terms. A dentist may practice a specialty of medicine, but he is not therefore a medical specialist. He may practice medicine, and yet not be a medical man. To become the latter, and to belong to the medical profession, he must have the medical degree, for that is the only qualification which medicine acknowledges. Having this, he belongs to the profession even though he does not practice at all.

I am a D. D. S.; my neighbor is in addition an M. D. Very well; he belongs to one more profession than I do. There is a distinguished dentist in New York who, in addition to both these, is also a D. D., and so belongs to yet another profession. He might declare that it is the duty of every dentist to do a certain amount of preaching, and to act as a teacher of the people. This is peculiarly the province of the clergy, and therefore he might insist that no man is fit to practice dentistry who has not gone through a thorough course in theology. Why need we quarrel over these things? Let each pursue his own course, so long as it is a reputable one, and call his practice what he will—dentistry simple, medico-dentistry, dental-medicine, or medicine pure.

